# 2-Amino-1,3-thia­zolium dihydrogen phosphate

**DOI:** 10.1107/S160053681104935X

**Published:** 2011-11-23

**Authors:** Irena Matulková, Jaroslav Cihelka, Ivan Němec, Michaela Pojarová, Michal Dušek

**Affiliations:** a Department of Inorganic Chemistry, Faculty of Science, Charles University in Prague, Hlavova 2030, 128 40 Prague 2, Czech Republic; b Institute of Physics of the ASCR, Na Slovance 2, 182 21 Prague 8, Czech Republic

## Abstract

In the title compound, C_3_H_5_N_2_S^+^·H_2_PO_4_^−^, the dihydrogen phosphate anions form infinite chains along [001] *via* short O—H⋯O hydrogen bonds. The 2-amino­thia­zolium cations inter­connect these chains into a three-dimensional network by short linear or bifurcated N—H⋯O and weak C—H⋯O hydrogen bonds.

## Related literature

For metal complexes of 2-amino­thia­zole and its derivatives used in medicine, see: De *et al.* (2008 ▶); Aridoss *et al.* (2009 ▶); Cukurovali *et al.* (2006 ▶); Franklin *et al.* (2008 ▶); Li *et al.* (2009 ▶); Alexandru *et al.* (2010 ▶); Mura *et al.* (2005 ▶). For the use of 2-amino­thia­zole in the decontamination of aqueous media or ethanol fuel, see: Cristante *et al.* (2006 ▶, 2007 ▶); Takeuchi *et al.* (2007 ▶). For uses of 2-amino­thia­zole and its derivatives as anti­corrosive films, see: Ciftci *et al.* (2011 ▶); Solmaz (2011 ▶). For non-linear optical properties and for structural properties of closely related compounds, see: Yesilel *et al.* (2008 ▶); Matulková *et al.* (2007 ▶, 2008 ▶, 2011*a* ▶,*b* ▶).
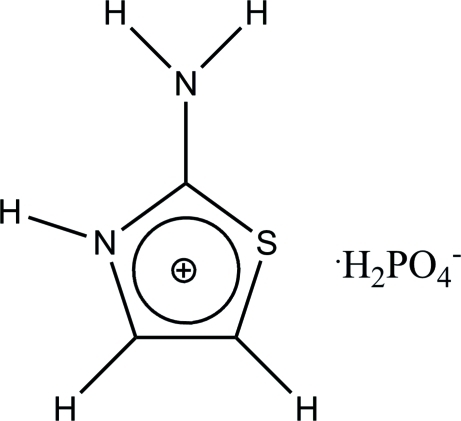


## Experimental

### 

#### Crystal data

C_3_H_5_N_2_S^+^·H_2_PO_4_^−^
*M* *_r_* = 198.14
Monoclinic, 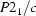

*a* = 9.7581 (2) Å
*b* = 9.8826 (2) Å
*c* = 8.2794 (1) Å
β = 90.680 (2)°
*V* = 798.37 (2) Å^3^
*Z* = 4
Cu *K*α radiation
μ = 5.35 mm^−1^
*T* = 120 K
0.47 × 0.17 × 0.13 mm

#### Data collection

Agilent Xcalibur Atlas Gemini ultra diffractometer
Absorption correction: multi-scan (*CrysAlis PRO*; Agilent, 2010 ▶) *T*_min_ = 0.453, *T*_max_ = 1.000
7670 measured reflections
1419 independent reflections
1389 reflections with *I* > 2σ(*I*)
*R*_int_ = 0.025

#### Refinement

*R*[*F* ^2^ > 2σ(*F* ^2^)] = 0.027
*wR*(*F* ^2^) = 0.070
*S* = 1.07
1419 reflections
100 parameters
H-atom parameters constrained
Δρ_max_ = 0.30 e Å^−3^
Δρ_min_ = −0.37 e Å^−3^

### 

Data collection: *CrysAlis PRO* (Agilent, 2010 ▶); cell refinement: *CrysAlis PRO*; data reduction: *CrysAlis PRO*; program(s) used to solve structure: *SHELXS97* (Sheldrick, 2008 ▶); program(s) used to refine structure: *SHELXL97* (Sheldrick, 2008 ▶); molecular graphics: *PLATON* (Spek, 2003 ▶); software used to prepare material for publication: *publCIF* (Westrip, 2010 ▶).

## Supplementary Material

Crystal structure: contains datablock(s) I, global. DOI: 10.1107/S160053681104935X/im2339sup1.cif

Structure factors: contains datablock(s) I. DOI: 10.1107/S160053681104935X/im2339Isup2.hkl

Supplementary material file. DOI: 10.1107/S160053681104935X/im2339Isup3.cml

Additional supplementary materials:  crystallographic information; 3D view; checkCIF report

## Figures and Tables

**Table 1:** Hydrogen-bond geometry (Å, °)

*D*—H⋯*A*	*D*—H	H⋯*A*	*D*⋯*A*	*D*—H⋯*A*
N1—H1*N*1⋯O2^i^	0.87	1.96	2.815 (2)	167
N1—H2*N*1⋯O1^ii^	0.80	2.31	3.076 (2)	162
N1—H2*N*1⋯O2^ii^	0.80	2.56	3.194 (2)	137
N2—H1*N*2⋯O3^i^	0.99	1.73	2.726 (2)	175
O1—H1*O*1⋯O2^iii^	0.90	1.61	2.504 (2)	176
O4—H1*O*4⋯O3^iv^	0.94	1.65	2.593 (2)	179
C2—H1*C*2⋯O4^v^	0.93	2.40	3.268 (2)	155

Symmetry codes: (i) 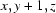; (ii) 
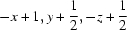
; (iii) 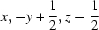; (iv) 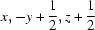; (v) 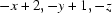.

**Fig. 1.**
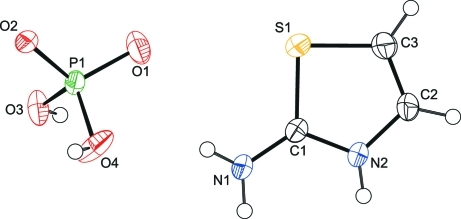
The molecular structure of 2-aminothiazolium dihydrogen phosphate. Displacement ellipsoids are drawn at the 50% probability level.

**Fig. 2.**
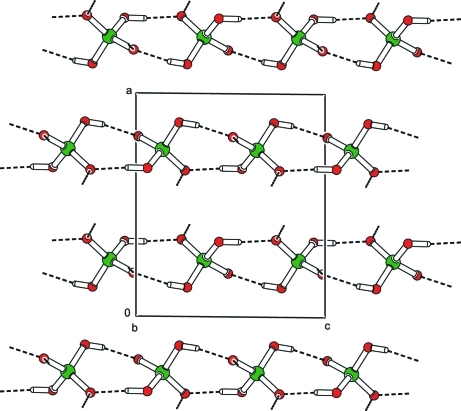
Packing scheme of the anions in the crystals of 2-aminothiazolium dihydrogen phosphate (projection to ac plane). Dashed lines indicate the hydrogen bonds.

**Fig. 3.**
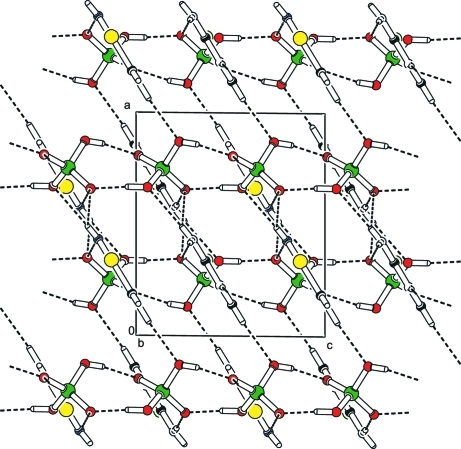
Packing scheme of the structure of 2-aminothiazolium dihydrogen phosphate (projection to ac plane). Hydrogen bonds are indicated by dashed lines.

## supplementary crystallographic information

### Comment

2-Aminothiazole and its derivatives have been investigated as potential compounds for the modification of TiO_2_ or SiO_2_ particles used for the sorption and photocatalytic reduction of Hg(II) (Cristante *et al.*, 2006) or phenol (Cristante *et al.*, 2007) in aqueous solutions. 2-Aminothiazole can be also used for electrode modification (Ciftci *et al.*, 2011; Solmaz, 2011) or for the detection of metal impurities (Takeuchi *et al.*, 2007) in ethanol fuel.

Metal complexes of 2-aminothiazole and its derivatives have been studied for treatment of Alzheimers disease (Li *et al.*, 2009), antitumor activity (Alexandru *et al.*, 2010), and activity against leukemia (Mura *et al.*, 2005). Thiazole derivatives have been used as antioxidants (De *et al.*, 2008), antibacterial drugs (Aridoss *et al.*, 2009) and fungicides (Cukurovali *et al.*, 2006). Anti-inflammatory, analgesic and antipyretic activities were observed for thiazolyl and benzothiazolyl derivatives (Franklin *et al.*, 2008).

Only one salt, bis(2-aminothiazolium) squarate dihydrate (Yesilel *et al.*, 2008), was studied in detail for the extensive system of hydrogen bonds, which are very attractive not only in the biological and biochemical processes but also in the field of material and supramolecular chemistry.

The title salt was prepared during the research motivated by the study of salts or cocrystals of the highly related aminotriazoles (Matulková *et al.*, 2011*a*, 2008, 2007) and 2-aminothiazole (Matulková *et al.*, 2011*b*), while searching for materials with potential non-linear optical properties. Unfortunately, the title salt, 2-aminothiazolium dihydrogen phosphate (Fig. 1), crystallizes in the monoclinic system in the centrosymmetric space group *P*2_1_/*c*, which excludes the existence of the second order non-linear optical properties. The crystal structure of the title compound is based on chains of anions interconnected *via* two O—H···O hydrogen bonds with donor-acceptor distances 2.504 (2) and 2.596 (2) Å. Chains are interconnected by 2-aminothiazolium(1+) cations *via* N—H···O (2.728 (2)–3.202 (3) Å) and weak C—H···O (3.271 (3) Å) hydrogen bond interactions into a three-dimensional network. Each cation interacts with three anionic chains by means of two linear hydrogen bonds towards one of the chains, one linear hydrogen bond to another chain and one bifurcated hydrogen bond to the third chain (Fig. 3). The anionic chains are oriented along the axis *c* (see Fig. 2).

### Experimental

Crystals of the title compound were obtained from a solution of 1.0 g of 2-aminothiazole (97%, Aldrich) and 0.67 ml of phosphoric acid (85%, Lachema) in 200 ml of water. The solution was left to crystallize at room temperature for several weeks. The colourless crystals obtained were filtered off, washed with methanol and dried in vacuum desiccator over KOH.

### Refinement

H atoms attached to C atoms were calculated in geometrically idealized positions, C*sp*^2^ - H = 0.93 Å. The positions of H atoms attached to O and N atoms were localized in difference Fourier maps. All hydrogen atoms were constrained to ride on their parent atoms during refinement, with *U*_iso_(H) = 1.2 *U*_eq_(pivot atom).

### Crystal data

**Table:**

C_3_H_5_N_2_S^+^·H_2_PO_4_^−^	*F*(000) = 408
*M**_r_* = 198.14	*D*_x_ = 1.648 Mg m^−^^3^
Monoclinic, *P*2_1_/*c*	Cu *K*α radiation, λ = 1.5418 Å
Hall symbol: -P 2ybc	Cell parameters from 6931 reflections
*a* = 9.7581 (2) Å	θ = 4.5–66.8°
*b* = 9.8826 (2) Å	µ = 5.35 mm^−^^1^
*c* = 8.2794 (1) Å	*T* = 120 K
β = 90.680 (2)°	Plate, colourless
*V* = 798.37 (2) Å^3^	0.47 × 0.17 × 0.13 mm
*Z* = 4	

### Data collection

**Table:**

Agilent Xcalibur Atlas Gemini ultra diffractometer	1419 independent reflections
Radiation source: Enhance Ultra (Cu) X-ray Source	1389 reflections with *I* > 2σ(*I*)
mirror	*R*_int_ = 0.025
Detector resolution: 10.3874 pixels mm^-1^	θ_max_ = 66.9°, θ_min_ = 4.5°
Rotation method data acquisition using ω scans	*h* = −11→11
Absorption correction: multi-scan (*CrysAlis PRO*; Agilent, 2010)	*k* = −11→11
*T*_min_ = 0.453, *T*_max_ = 1.000	*l* = −9→7
7670 measured reflections	

### Refinement

**Table:**

Refinement on *F*^2^	Primary atom site location: structure-invariant direct methods
Least-squares matrix: full	Secondary atom site location: difference Fourier map
*R*[*F*^2^ > 2σ(*F*^2^)] = 0.027	Hydrogen site location: inferred from neighbouring sites
*wR*(*F*^2^) = 0.070	H-atom parameters constrained
*S* = 1.07	*w* = 1/[σ^2^(*F*_o_^2^) + (0.0339*P*)^2^ + 0.5451*P*] where *P* = (*F*_o_^2^ + 2*F*_c_^2^)/3
1419 reflections	(Δ/σ)_max_ = 0.001
100 parameters	Δρ_max_ = 0.30 e Å^−^^3^
0 restraints	Δρ_min_ = −0.37 e Å^−^^3^

### Special details

**Table:**

Geometry. All e.s.d.'s (except the e.s.d. in the dihedral angle between two l.s. planes) are estimated using the full covariance matrix. The cell e.s.d.'s are taken into account individually in the estimation of e.s.d.'s in distances, angles and torsion angles; correlations between e.s.d.'s in cell parameters are only used when they are defined by crystal symmetry. An approximate (isotropic) treatment of cell e.s.d.'s is used for estimating e.s.d.'s involving l.s. planes.
Refinement. Refinement of *F*^2^ against ALL reflections. The weighted *R*-factor *wR* and goodness of fit *S* are based on *F*^2^, conventional *R*-factors *R* are based on *F*, with *F* set to zero for negative *F*^2^. The threshold expression of *F*^2^ > σ(*F*^2^) is used only for calculating *R*-factors(gt) *etc*. and is not relevant to the choice of reflections for refinement. *R*-factors based on *F*^2^ are statistically about twice as large as those based on *F*, and *R*- factors based on ALL data will be even larger. The hydrogen atoms were be localized from the difference Fourier map. Despite of that,all hydrogen atoms connected to C were constrained to ideal positions. The distance in N—H and O—H groups were left unrestrained. The isotropic temperature parameters of hydrogen atoms were calculated as 1.2**U*_eq_ of the parent atom.

### Fractional atomic coordinates and isotropic or equivalent isotropic displacement parameters (Å^2^)

**Table:**

	*x*	*y*	*z*	*U*_iso_*/*U*_eq_	
C1	0.66442 (17)	0.80237 (17)	0.1326 (2)	0.0234 (4)	
C2	0.85753 (17)	0.75229 (19)	−0.0086 (2)	0.0283 (4)	
H1C2	0.9367	0.7716	−0.0657	0.034*	
C3	0.81429 (18)	0.62710 (18)	0.0206 (2)	0.0301 (4)	
H1C3	0.8591	0.5492	−0.0132	0.036*	
N1	0.57066 (15)	0.87839 (15)	0.20091 (19)	0.0303 (4)	
H1N1	0.5829	0.9648	0.2168	0.036*	
H2N1	0.5099	0.8487	0.2540	0.036*	
N2	0.77265 (14)	0.85134 (15)	0.05485 (17)	0.0237 (3)	
H1N2	0.7837	0.9509	0.0454	0.028*	
O1	0.66404 (15)	0.33219 (15)	0.06047 (15)	0.0400 (4)	
H1O1	0.6636	0.3367	−0.0481	0.048*	
O2	0.65458 (14)	0.14664 (14)	0.25914 (15)	0.0338 (3)	
O3	0.80601 (12)	0.12283 (11)	0.01267 (14)	0.0243 (3)	
O4	0.87030 (12)	0.28418 (13)	0.23071 (15)	0.0301 (3)	
H1O4	0.8469	0.3189	0.3326	0.036*	
P1	0.74805 (4)	0.21447 (4)	0.14002 (5)	0.02120 (14)	
S1	0.66311 (4)	0.62775 (4)	0.13014 (5)	0.02763 (15)	

### Atomic displacement parameters (Å^2^)

**Table:**

	*U*^11^	*U*^22^	*U*^33^	*U*^12^	*U*^13^	*U*^23^
C1	0.0219 (8)	0.0251 (9)	0.0234 (9)	−0.0034 (6)	0.0016 (6)	−0.0005 (6)
C2	0.0210 (8)	0.0331 (9)	0.0310 (9)	0.0010 (7)	0.0049 (7)	−0.0035 (8)
C3	0.0220 (9)	0.0288 (10)	0.0398 (10)	0.0024 (7)	0.0051 (7)	−0.0049 (7)
N1	0.0277 (8)	0.0262 (8)	0.0373 (9)	−0.0053 (6)	0.0157 (7)	−0.0039 (6)
N2	0.0216 (7)	0.0245 (7)	0.0250 (7)	−0.0023 (5)	0.0050 (5)	−0.0015 (6)
O1	0.0515 (9)	0.0436 (8)	0.0247 (7)	0.0265 (7)	−0.0028 (6)	−0.0091 (6)
O2	0.0347 (7)	0.0371 (7)	0.0300 (7)	−0.0138 (6)	0.0155 (5)	−0.0113 (6)
O3	0.0287 (6)	0.0216 (6)	0.0227 (6)	0.0031 (5)	0.0089 (5)	0.0001 (4)
O4	0.0228 (6)	0.0444 (8)	0.0231 (6)	−0.0071 (5)	0.0070 (5)	−0.0071 (5)
P1	0.0201 (2)	0.0230 (2)	0.0207 (2)	0.00071 (15)	0.00549 (16)	−0.00254 (15)
S1	0.0252 (2)	0.0228 (2)	0.0351 (3)	−0.00293 (15)	0.00515 (18)	−0.00053 (16)

### Geometric parameters (Å, °)

**Table:**

C1—N1	1.317 (2)	N1—H2N1	0.7980
C1—N2	1.334 (2)	N2—H1N2	0.9934
C1—S1	1.726 (2)	O1—P1	1.564 (1)
C2—C3	1.330 (3)	O1—H1O1	0.8996
C2—N2	1.389 (2)	O2—P1	1.508 (1)
C2—H1C2	0.9300	O3—P1	1.505 (1)
C3—S1	1.741 (2)	O4—P1	1.562 (1)
C3—H1C3	0.9300	O4—H1O4	0.9413
N1—H1N1	0.8718		
			
N1—C1—N2	123.9 (2)	C1—N2—C2	113.9 (2)
N1—C1—S1	124.79 (13)	C1—N2—H1N2	119.0
N2—C1—S1	111.3 (2)	C2—N2—H1N2	127.1
C3—C2—N2	113.3 (2)	P1—O1—H1O1	117.0
C3—C2—H1C2	123.4	P1—O4—H1O4	113.5
N2—C2—H1C2	123.4	O3—P1—O2	115.19 (7)
C2—C3—S1	111.3 (1)	O3—P1—O4	108.11 (7)
C2—C3—H1C3	124.3	O2—P1—O4	110.24 (7)
S1—C3—H1C3	124.3	O3—P1—O1	110.61 (7)
C1—N1—H1N1	121.9	O2—P1—O1	106.73 (8)
C1—N1—H2N1	123.4	O4—P1—O1	105.54 (8)
H1N1—N1—H2N1	112.3	C1—S1—C3	90.21 (8)

### Hydrogen-bond geometry (Å, °)

**Table:**

*D*—H···*A*	*D*—H	H···*A*	*D*···*A*	*D*—H···*A*
N1—H1N1···O2^i^	0.87	1.96	2.815 (2)	167
N1—H2N1···O1^ii^	0.80	2.31	3.076 (2)	162
N1—H2N1···O2^ii^	0.80	2.56	3.194 (2)	137
N2—H1N2···O3^i^	0.99	1.73	2.726 (2)	175
O1—H1O1···O2^iii^	0.90	1.61	2.504 (2)	176
O4—H1O4···O3^iv^	0.94	1.65	2.593 (2)	179
C2—H1C2···O4^v^	0.93	2.40	3.268 (2)	155

Symmetry codes: (i) *x*, *y*+1, *z*; (ii) −*x*+1, *y*+1/2, −*z*+1/2; (iii) *x*, −*y*+1/2, *z*−1/2; (iv) *x*, −*y*+1/2, *z*+1/2; (v) −*x*+2, −*y*+1, −*z*.
